# A New Year's Appeal

**Published:** 1922-01

**Authors:** 


### A NEW YEAR'S APPEAL.

LAST MONTH we published a Christmas Appeal for the London Hospitals. We mentioned by name several that were especially in want and we indicated also the nature of those needs. At the same time we invited other hospitals, not mentioned in that list, to communicate with us and to let us know their chief needs so that we might give useful publicity to these. Below we print a list of those hospitals which have taken advantage of our offer, and we commend their wants to the kindness and consideration of our readers.

Christmas and New Year Appeals tend sometimes, unfortunately, towards hysteria. None denies that our hospitals need and deserve all the help that they can obtain, and there is none who does not appreciate the work they have done for so long in alleviating sickness and in preventing disease. Many of us have personal knowledge that home is the worst possible place in which to be ill, and that sickness in hospital is more tolerable than it is elsewhere; but that is no reason for the hysterical outbursts that are seen occasionally in the lay Press regarding the poverty of our hospitals. We publish in another column a bitter, but not altogether undeserved, rebuke from a contemporary, the "Pall Mall and Globe." It is necessary in these days for our charitable institutions to advertise their needs; but unbalanced and emotional propaganda, undertaken on their behalf by the lay Press, and being, as it is sometimes, little short of political propaganda, does disservice and even actual harm to the charitable cause. No hospital desires to be associated with politics or to be used as a pawn in any political game, and we are sure that hospitals do not really welcome these champions who take up on their behalf such controversial cudgels. In the year before us we need especially faith and hope in our high cause of charity; and to appeal to the hearts of the people rather than to their political creeds. We believe finally that as the result of hard work, care and co-ordination, the hospitals in London and in the country as a whole will surmount their present difficulties, and that by the next New Year they will be able to look back on the present time with that satisfaction which the fighter experiences when he has worthily overcome his trials.

### HOSPITALS WITH OVER 150 BEDS.

### Charing Cross Hospital, Strand, W.C. 2.

Situated in the busiest centre of the busiest city in the world, it treats every year more than 12,000 casualties. Owing to the generosity of the public and the enforcement of rigid economy, the Hospital is out of debt, but a forward programme has been arranged for 1922, for which purpose a sum of £38,000 is urgently required. One of the greatest needs is the building and equipment of a Maternity Ward. Another crying need is additional accommodation for the Nursing and Domestic Staffs.

### Guy's Hospital, S.E. 1.

The special needs of this charity are: (1) Increased income from voluntary sources to bridge the gap between assured income and expenditure, and particularly to make good the loss of income involved in the recent unavoidable expenditure of capital on long-deferred improvements. (2) Generous help to replace the capital cost of the new Nurses' Extension Departments of Massage and Electricity and Children's Ward, and to enable these and a further provision for Specialisms to be completed and adequately equipped.

An appeal is being circulated and a systematic canvass made among firms and individuals having interests in the neighbourhood of the Hospital and in the area from which a large part of the patients are drawn. A small charge is made (except to the very poor) for medicines supplied to persons attending the Out-patient Departments. Every In-patient receives on quitting the Hospital a brief statement of the cost of treatment and an appeal to contribute to the funds in accordance with his means, and a considerable proportion gladly respond. The organisation of contributions from the working classes is now being studied with King Edward's Hospital Fund and other hospitals with a view to formulating an agreed scheme for appealing to weekly workers.

### Hospital for Consumption and Diseases of the Chest, Brompton, S.W. 3.

This Hospital was the principal centre for the treatment of men invalided from both Services suffering from consumption, the numbers admitted to both the Hospital and the Sanatorium at Frimley running into some thousands. There are long waiting lists and more patients could be treated if additional funds could be provided for the Sanatorium, where there is ample accommodation for extension. Although the Hospital has done much to help itself by a plan for obtaining contributions from the patients, their employers and other authorities, which has now been developing for some years, there remains a wide margin to be made up.

### King's College Hospital, Denmark Hill, S.E. 5.

This, the first great teaching Hospital, began 1921 with a total debt of £100,000, including the deficit of £20,000 on the work of 1920. By keeping open and in use the 400 beds this enormous debt was being added to at the rate of at least £1,000 per week, and after very careful consideration and with the utmost reluctance the Committee of Management were compelled to close seven wards containing 160 beds. The annual expenditure of the Hospital with its 400 beds and all the special departments in full use is about £131,000, and the total annual income which can be looked upon as assured is but £51,000. The most pressing needs then, are: (1) £100,000 to liquidate the Debt; (2) £80,000 a year additional income to make it possible to re-open and maintain the 160 closed beds. The closing of these beds is inflicting much suffering upon large numbers of sick men, women and children. As long as the beds remain closed more than 3,000 persons each year must be refused that treatment which they so urgently need. Again, if the beds remain closed indefinitely, the training of doctors and nurses will be seriously hindered.

A great effort is being made to induce all those in the district served by the Hospital who can do so to subscribe at least a guinea or more each year. At the same time, workers of all classes are being invited to contribute some definite amount, not less than 2d. each per week, towards the funds of the Hospital.

### Royal National Orthopædic Hospital, Great Portland Street, W.

The Hospital's present buildings are new and up-to-date, but the 200 beds which are available are hopelessly inadequate. More than 1,000 children are on the lists waiting admission. Many may have to wait three or four years for their turn. Moreover, children, grown-up cripples, and men disabled in the war, come to the Out-patients' Department; the attendances have grown from 11,000 in 1908 to 80,000 in 1920. The Hospital appeals for £200,000.

(1) To extend the present buildings on an adjoining site already acquired, and so provide:—

(*a*) Adequate space for dealing with Out-patients; (*b*) accommodation for paying patients who are not rich enough to pay the high fees of nursing homes; (*c*) the foundation of a School of Orthopædics; (*d*) space for the extension of the workshops for making surgical appliances and boots; (*e*) accommodation for additional medical and nursing staffs.

(2) To form a large Country Branch with 200 to 300 beds for children.

**A New Year Appeal for Hospitals—(*cont.*).**

**Seamen's Hospital Society.**

This old Corporation now comprises three Hospitals—the Dreadnought at Greenwich; the Albert Dock Hospital; and the Hospital for Tropical Diseases—a Sanatorium and a Convalescent Home.

The mariner, stricken with accident or disease, comes to the various establishments of the Society from every port in the Kingdom, almost from every port in the world. Over 18,000 patients are treated annually. To the Hospital for Tropical Diseases at Endsleigh Gardens is attached the London School of Tropical Medicine, which has made rapid progress since its removal to town two years ago. King George's Sanatorium for Sailors was opened in July last, and more than half the beds are already occupied. The expenditure of the Society is upwards of £70,000, and an earnest appeal for help is made to all who recognise what we owe to the mariner and what we owe to the pioneers in tropical medicine.

**University College Hospital, Gower Street, W.C. 1.**

This Hospital is in need of £20,000, not to pay off debts, but in order that its work may continue on its past scale and that its future development may be maintained. It must be borne in mind that attached to this Hospital is one of our premier Medical Schools, and progress is the keynote of this policy. In endeavouring to increase its reliable funds the Hospital is endeavouring to give something in return to subscribers, and in addition to offering them the facilities of the Hospital it is pointed out to all that it is their duty to subscribe in order to provide those better and more skilful Doctors and Nurses of to-morrow, who shall tend and care for those who follow after us. Only by the non-curtailment of the work of a Hospital like this can that ceaseless research be carried on which must not be allowed to falter for one minute if the secret of such dread diseases as cancer and tuberculosis are ever to be discovered.

This Hospital makes a very real and genuine appeal for the first time for 90 years to increase its income by £20,000 per annum.

**Westminster Hospital, Broad Sanctuary, S.W. 1.**

£15,000 per annum is needed, in addition to the regular income from subscriptions and investments; and £50,000 for essential alterations and improvements to the present Hospital building.

The Committee intends to appeal to the general public, and particularly to those who use the Hospitals, to make regular contributions, however small. A systematic quarterly house-to-house collection is being organised in the area served by the Hospital. Some form of weekly collections from the wage-earning classes in the district is to be put into operation. Patients will be invited to contribute according to their means while under treatment. Expenditure is decreasing and income is increasing, and it is hoped that the deficit next year will not be so great.

**The Middlesex Hospital and Cancer Charity.**

In order to meet the increased cost of maintenance for 1921 it has been necessary to obtain an overdraft from the bankers of £35,000 and to pledge to them as security every realisable asset which the Hospital possesses. In addition to the continuance of, and improvements in, the system of contributions by patients towards maintenance, the main policy of the Hospital for 1922 will be directed towards securing larger permanent support by a thoroughly organised scheme for collections from firms in the neighbourhood of the Hospital. This scheme aims at educating both employees and employers as to the real functions of a Hospital, and interesting them in its many-sided activities. The scheme was started some little time ago, and gives promise of appreciable success.

**London Lock Hospital, 283, Harrow Road, W. 9.**

The estimated liabilities of this old-established Hospital, including bank overdraft and bills owing to tradesmen, is £10,781. An earnest Christmas appeal is being made for donations to clear off this big deficit. The patients in the wards of this Hospital are not well-paid employees, but form the saddest and most neglected portion of the population of our country. A large number of beds are devoted to babies and young children suffering through no fault of their own, and no less than 253 babies have been born in the Hospital free of venereal disease, owing to the special treatment devised by the Hon. Surgeons. The London Lock Hospital is co-operating with ten other Hospitals and Charities in connection with a Christmas Carnival and Children's Fair at the Royal Albert Hall from December 26 to January 4. Those who feel they cannot send a donation to the Christmas Appeal are earnestly asked to buy tickets for this Carnival.

**The London Hospital, E. 1.**

A recent incident fairly illustrates the position of England's biggest hospital—"The London"—at the close of 1921. An American visitor, whose wife had been cured in very dramatic circumstances by one of the hospital's surgeons, sent a cheque for £5,000 as a thank-offering and promised a further £5,000 provided that the hospital would raise £10,000 by December 31—all the money to be ear-marked for the much-needed improvement of the hospital's maternity work. But what prospect is there to-day of "making good" this splendid offer, when the Hospital's bare existence is from day to day, when every penny it can raise is needed for daily bread? Two hundred beds have been and still are closed, which means 4,000 sick people denied their chance in a year; half the Out-Patient work is suspended; there is a debt of £40,000 and the wear and tear of seven years to be made good! Even allowing for large sums from patients' payments and the aid of the great funds, the Hospital requires for bare efficiency quite £75,000 a year beyond its normal support. Unless further catastrophic cutting in all directions is to be made, it appears that the way of salvation and stabilisation must be sought in some form of insurance, of guaranteed treatment or freedom from hospital charges, in return for guaranteed contributions.

**Metropolitan Hospital, Kingsland Road, E. 8.**

Through a large amount of additional work this hospital is able to show a more satisfactory statement of ordinary income and expenditure than last year, when trade conditions were better. The deficit last year was £6,443; this year it will not largely exceed £2,000. Efforts have been largely turned towards increasing the contributions from work-people, with encouraging success. An Aid Society is collecting funds for the upkeep of certain Wards. An Appeals Committee is preparing a big scheme of a house-to-house collection to be launched in the district early in 1922.

The Hospital needs many improvements. The kitchens are unsatisfactory, and a large amount of new equipment will soon be required. The storage accommodation for provisions is also most inadequate. Another urgent requirement is a satisfactory Nurses' Home. At present there is no prospect of paying off even a portion of the mortgage of £10,000 on the Hospital buildings. The daily average number of in-patients has increased in 1921 to 115, against 108 last year.

### HOSPITALS WITH UNDER 150 BEDS.

**Chelsea Hospital for Women, Arthur Street, S.W. 3.**

This institution is making every effort to effect two main objects: (1) The building of a Nurses' Home, which will free for patients 40 beds now used by the staff; and (2) the increase of its income to meet its expenditure. As regards (2), there is a deficiency in what may be called reliable income of £2,000 per annum, and to assist in making this good, legacies are greatly needed. £12,500 more is required for the Nurses' Home. When the additional beds in the Hospital can be used for patients, many of them will be available for those who in the past would have gone into nursing-homes, but whose reduced means now render that course impossible. Their fees should, with the resources now under the consideration of the King Edward's Fund, be successful in re-establishing the financial position.

**The "Evelina" Hospital, Southwark Bridge Road, S.E. 1.**

The largest Children's Hospital in South London. £5,000 is owing to the bankers; the deficit for the year will be not less than £6,000; moreover, about £5,000 is urgently required

**A New Year Appeal for Hospitals—(*cont.*).**

for the thorough renovation of the Hospital, which work is many years overdue. £10,000 is, therefore, urgently wanted; further, it is imperative to increase the annual income by at least £4,000 to meet the ordinary expenditure, which now amounts to about £14,000 a year. Owing to its situation in the poorest district of South London, the "Evelina" Hospital is rarely seen by the wealthy and suffers accordingly.

**The Hospital for Epilepsy and Paralysis, Maida Vale, W.**

A pioneer in the matter of patients' payments, this hospital stands most in need of £5,000 to make up the sum of £10,000 required to extend the Out-Patient Department, which is rapidly becoming an outdoor department. A large number of patients have to wait out of doors, there being no accommodation for them.

**The Infants' Hospital, Vincent Square, Westminster, S.W.**

This hospital has now reached the limit of its resources. It has no funds, is deeply in debt, and is unable to borrow any more money. Since 1914 its income has been insufficient to meet the increased cost of maintenance; and the Committee are now faced with the necessity of closing it unless increased support can be obtained. An effort is being made to reduce expenditure by rigorous economy, and to increase income by getting new supporters. Unless this is successful the important work of the hospital must shortly come to an end.

**The Miller General Hospital for South-East London, Greenwich Road, S.E. 10.**

Of the extension scheme the most pressing need is a further ward block and a portion of the Nurses' Home. The site has been purchased and there is about £20,000 in hand. With another £30,000 a beginning could be made. South-East London has been sadly overlooked in the past from the point of view of hospital accommodation. There are now 100 beds, where ten years ago there were only 25, but at least three times as many are wanted.

**Poplar Hospital for Accidents, East India Dock Road, E. 14.**

An important part of this hospital's work is to deal with the many serious accidents which occur among the thousands of workmen employed in the docks and factories of the East End. It is appealing for £20,000—£5,000 towards current expenses and £15,000 to complete its rebuilding scheme, which provides for:—(1) The rebuilding of the West end of the Hospital, including the Casualty Department, with a new road entrance; (2) an X-Ray Operating Room; (3) an Operating Room for Out-patients; (4) a Massage Room; (5) an additional Ward for Children; (6) a new Nurses' Home; and (7) additional Rooms for Staff and Patients. The Hospital has never been in debt. It has, therefore, invited tenders for this work in sections, and will only commit itself to the extent that funds in hand permit.

**Queen Charlotte's Lying-in Hospital, Marylebone Road, N.W.**

During the past year some much-needed reconstruction has been carried out. Most of the smaller wards have been converted into larger wards; the ventilation has been much improved, and lavatory basins with hot and cold water have been fitted to every ward. Other improvements still remain to be made. The erection of a new Out-Patient Department is impossible until the financial position greatly improves. Meanwhile, the important ante-natal and child-welfare work, which is constantly increasing, is carried on in the temporary Out-Patient Department. The hospital is greatly in need of additional support, for while the annual expenditure is now little short of £20,000, the income which can be relied upon is not much more than £10,000.

**The Queen's Hospital for Children, Bethnal Green, E.2 (Late North-Eastern Hospital for Children).**

This hospital is now nearly £10,000 in debt to bankers and tradesmen, and had to provide another £3,000 to meet expenses of maintenance up to December 31, 1921. The wards (providing 134 beds here and 36 at Bexhill) are always taxed to the utmost, and every department of the hospital is working at high pressure. To turn the children and anxious mothers away seems impossible, yet the dreaded position is near when the closing of wards and the partial closing of the Out-patient and Casualty-room doors may be unavoidable. The parents are contributing on a voluntary basis according to their means, and the people throughout this part of London are giving the hospital assistance by the organised collection of small sums.

**The Royal Waterloo Hospital for Children and Women, Waterloo Road, S.E. 1.**

There is an accumulated deficit on the General Purposes Account of approximately £5,000. The much-needed extension, to consist of a new Nurses' Home, Isolation and an enlarged Out-patient Department, has had to be temporarily suspended at the first floor level through lack of funds. Moral pressure is brought to bear upon all patients to contribute according to their means, and an increasing amount is expected from this source. In addition, the hospital keeps in touch with all old patients, and an increasing number month by month send small contributions and undertake collecting cards. A weekly local collection by the nursing staff has brought in good results, as have also the new posters and collecting-boxes.

**St. Andrew's Hospital, Dollis Hill, London. N.W. 2.**

Help is needed for (1) The maintenance of the hospital; (2) the paying off of the deficit of about £1,200 on current account; (3) the extinction of the building debt of £668; (4) the completion of the furnishing and equipment of the hospital and the chapel; (5) the endowment of the hospital; (6) the completion of the building. Special needs are an electric department and a mortuary.

**St. Mark's Hospital, City Road, E.C. 1.**

Requires £7,000 from voluntary contributions to help meet its ordinary expenditure and pay off a loan. X-Ray and Pathological departments are required to help in the fight against the scourge of rectal cancer. The increasing number of cancer cases amongst women admitted to this hospital may necessitate the building of a special cancer ward. The sum of £3,000 may be required for these additions.

**Samaritan Free Hospital for Women, Marylebone Road, N.W. 1.**

A debt to the bankers of £2,500, outstanding tradesmen's accounts since September amounting to over £2,000, and all realisable securities pledged, is the disastrous position in which the hospital is placed. Help is immediately needed to enable the Committee to continue this most essential work. A very cordial invitation is extended to any reader of the HOSPITAL AND HEALTH REVIEW to visit this hospital.

**South-Eastern Hospital for Children, Sydenham.**

Is finishing the year with a debt of some £1,000, chiefly through extra expenditure in doing work which had to be neglected during the war years. The whole of the interior has been redecorated, central heating has been furnished, and other necessary improvements made, bringing the in-patients' department up to date. Hopes, with the aid of the Local Hospital Welfare now working in the district, to receive grants. Will celebrate its jubilee next year, when every effort will be made to raise £10,000 towards the necessary rebuilding of the out-patients' department, for which plans have been sanctioned by King Edward's Fund. Several functions are being prepared with which it is hoped to celebrate the important anniversary and bring in the needed funds.

**South London Hospital for Women, South Side, Clapham Common, S.W.**

Some friends of the hospital recently presented to the institution the freehold of an adjoining property, with the object of providing 30 additional beds for surgical cases, together with necessary accommodation for nursing and domestic staffs. It is anticipated that the cost of altering and adapting the existing premises and connecting with the main hospital buildings by a sub-way will amount to £13,500, and the required equipment to a further £3,000. Toward this capital expenditure a sum of £12,000 is already guaranteed. A considerable proportion of the increased annual expenditure will be met by patients' payments, the hospital having, since its inception, expected patients to contribute in accordance with their means; while an Auxiliary Workers' Association with four branches in South London has recently been formed with the object of raising money to meet the balance. The building of a new Out-patients' Department on a site in Newington Butts, presented for this purpose, remains in abeyance, pending the raising of the necessary funds.

**A NEW YEAR'S APPEAL—** (*cont. from p.* 111).

**The Victoria Hospital for Children, Tite Street, Chelsea, S.W. 3.**

Will need an additional £5,000 a year in new money, besides £8,000 to wipe off the existing bank deficit, if it is to pay its way during 1922. A system of voluntary payments by patients' parents has been introduced with marked success, adding over £1,100 to its revenue in 1921. Indigent cases are still treated free. An up-to-date Physio-Therapy department for saving deformed children in early and preventable stages from becoming life-long cripples will be opened early in the New Year. The heating plant throughout the institution requires renewal, and the scheme for installing central heating is at present shelved through lack of funds.

**Alexandra Hospital for Children with Hip Disease, 1, Upper Woburn Place, W.C.1.**

£3,000 is urgently needed to maintain the beds for the little patients, most of them children of our sailors and soldiers.

